# Do USMLE steps, and ITE score predict the American Board of Internal Medicine Certifying Exam results?

**DOI:** 10.1186/s12909-020-1974-3

**Published:** 2020-03-18

**Authors:** Supratik Rayamajhi, Prajwal Dhakal, Ling Wang, Manoj P. Rai, Shiva Shrotriya

**Affiliations:** 1 Department of Medicine, Michigan State University, 788 Service Road, Room B301 Clinical Center, East Lansing, MI 48824 USA; 2 Division of Oncology and Hematology, Department of Internal Medicine, University of Nebraska Medical Center, Omaha, NE USA; 3 Fred and Pamela Buffett Cancer Center, University of Nebraska Medical Center, Omaha, NE USA

**Keywords:** USMLE Step 1, USMLE Step 2CK, USMLE Step 3, 3rd year in-training exam results, American board of internal medicine certifying exam (ABIM-CE)

## Abstract

**Background:**

To evaluate if United States Medical Licensing Examination (USMLE) Step 1, USMLE Step 2 CK, USMLE Step 3, and residency third-year in-service training exam (ITE) scores predict the results of American Board of Internal Medicine Certifying Exam (ABIM-CE).

**Methods:**

We performed a retrospective review of USMLE Step 1, USMLE Step 2 CK, USMLE Step 3, third-year residency ITE scores and ABIM-CE results of IM residents at our residency program from 2004 through 2017. Statistical analysis was perfrormed using Pearson correlation coefficient, and logistic regression to assess the relationship between USMLE Step 1, USMLE Step 2CK, USMLE Step 3, 3rd year ITE scores and ABIM-CE results. We used Multivariate logistic regression to predict pass or fail results in ABIM-CE based on USMLE and third-year ITE test scores controlling for other covariates.

**Results:**

Among 114 Internal Medicine MD residents included in the study, 92% (*n* = 105) passed the ABIM-CE. The OR of passing ABIM-CE was 2.70 (95% CI = 1.38–5.29), 2.31 (95% CI = 1.33–4.01), and 1.63 (95% CI = 0.81–3.29) with a ten-point increase in USMLE Step 1, USMLE Step 2 CK and USMLE Step 3 scores respectively. The OR of ABIM-CE passing chance was 2.96 (95% CI = 0.95–9.20), with a ten-point increase in the average score of the above three exams. A 5 % increase in ITE percentage raised the likelihood of passing ABIM-CE (OR 2.92, 95% CI 1.15–7.38). All residents who failed ABIM-CE had Step 1 scores < 220. Among 31 residents with Step 2 CK score < 220, 20% (*n* = 6) failed ABIM. Similarly, 9% of residents with USMLE Step 3 score < 220 failed ABIM-CE; all residents who failed had scored < 220. The probability curve predicted that the chance of passing ABIM- CE was around 80% with USMLE scores greater than 200 and increased to almost 100% with USMLE scores of 250 or more.

**Conclusion:**

USMLE Step 1, USMLE Step 2 CK, and third-year ITE scores can predict the chances of passing ABIM-CE. The third-year ITE score has a higher preditive value compared to USMLE Step 1 and USMLE Step 2 scores. USMLE Step 1 scores more predictive of ABIM-CE results compared to USMLE Step 2CK scores. Thus, residency programs can identify internal medicine residents at risk of failing ABIM-CE and formulate interventions at an early stage during residency training. Measures such as enrolling them in question banks or board review courses can be helpful in improving their chances of passing ABIM-CE.

## Background

The assessment of a resident's competency during training is a complex process. Residency programs perform a twice yearly evaluation of the six core competencies identified by Accreditation Council for Graduate Medical Education (ACGME) to improve the overall performance of the residents [1]. American Board of Internal Medicine (ABIM) uses various factors to test the skills of the physicians and their ability to deliver high-quality care. The factors include fulfillment of the graduate medical education training requirements, demonstration of clinical competence in patient care, and passing the American Board of Internal Medicine Certifying Exam (ABIM-CE) [2]. ABIM-CE is administered once every year for residents who complete their training. ABIM-CE results are important for both the residents and their residency programs. In order for the residency programs to maintain their ACGME accreditation, ACGME requires a 80% pass rate in ABIM-CE from the first-time takers of the exam in the latest three-year period [3].

Various tools are used by IM residency programs to select candidates likely to perform well in ABIM-CE to maintain ABIM-CE success rate. United States Medical Licensing Examination (USMLE) scores are commonly used by residency programs to screen residency applicants [4–6]. During the residency training, the annual in-service training exam (ITE) serves as a tool to assess the medical knowledge of the residents. A national standardized ITE was developed for various specialties, including IM, to provide feedback to the residents and the training program [7]. By assessing the knowledge gap (the deficit in the knowledge that can be improved to achieve better scores), ITE exams allow the residents to understand the areas in need for improvement. Also, the ITE exam score is used by the training programs to evaluate the residents readiness for ABIM-CE and the residents ability pass ABIM-CE [7].

 Previously, several studies have reported variable degrees of correlation between USMLE and ITE scores [5, 6, 8, 9]. A few studies reported an association between USMLE scores and IM-ITE performance [10, 11]. A modest correlation between failing USMLE Step 1 and scoring at the bottom quartile in ITE scores with risk of failing ABIM-CE was reported by Kay et al. [12]. In our study, we analyze whether USMLE Step 1, USMLE Step 2 CK, USMLE Step 3, and residency third year ITE scores correlated with the results of ABIM-CE.

## Methods

### Study sample and characteristics

All residents enrolled in our IM residency training program from 2004 through 2017 were included. The collected data included gender; type of degree (MD versus DO); medical school country (American Medical Graduate [AMG] versus International Medical Graduate [IMG]); scores in USMLE Step 1, USMLE Step 2 CK, USMLE Step 3, 3rd year ITE results; gap (number of years) between graduation from medical school and start of residency (referred to as ‘the gap’); and ABIM-CE results (Pass versus Fail). The number of osteopathic residents (DO) was considerably less than allopathic residents (MD), and they had taken COMLEX exams instead of USMLE Steps so DO residents were excluded. Also, the MD residents without complete data were excluded. Thus, after excluding DO residents (*n* = 18) and MD residents with incomplete data (*n* = 9), the remaining MD residents were included in the analysis.

Scores of ITE from the third year of residency, the exam closest to ABIM-CE in the timeline, were used in our analysis because third-year ITE scores reflect the readiness of the residents to take ABIM-CE.

### Statistical analysis

We presented all continuous data as mean (SD), and categorical data as N(%), Statistical analyses were based on scaled scores. We used the Pearson correlation coefficient to assess the correlation between USMLE Step 1, USMLE Step 2 CK, USMLE Step 3, and third-year ITE scores. We performed logistic regression to evaluate the relationship between various scores separately and pass or fail results in ABIM-CE. Also, we performed multivariate logistic regression to examine the relationship between the scores and the ABIM-CE results controlling for other confounding variables such as gender, medical school country, the gap between medical school and residency program. Significance was set at *p* < 0.05. We used SAS 9.4 software (SAS Institute Inc., Cary, North Carolina) to perform statistical analysis.

### Ethics approval

We received an exemption from the approval of Michigan State University Human Research Protection Program – MSU institutional board review (IRB# × 16-029e). We did not obtain consent from individual graduates, the data is de-identified, and the ethics committee approved this procedure.

## Results

We included a total of 114 MD residents in the study; 92% (*n* = 105) passed the ABIM-CE. Table 1 shows the characteristics of the residents, along with their mean scores in different exams. Table 2 shows the mean USMLE Step 1, USMLE Step 2CK, USMLE Step 3 and 3rd year ITE scores of based on ABIM-CE results.

**Table 1:** Demographic characteristics and examination scores

Gender (n, %)	
Female	34 (29.8)
Male	80 (70.2)
Medical School Country (n, %)	
United States or Canada	17 (14.9)
International	97 (85.1)
Gap between medical school graduation and start of residency (years, mean (range))	3.1 (0–17)
USMLE Scores (mean (SD))	
Step 1	230 (19)
Step 2	230 (21)
Step 3	208 (13)
Mean of Step 1, Step 2 and Step 3	222 (14)
Third-year ITE percentage (mean (SD))	68 (7)
ABIM-CE results (n, %)	
Pass	105 (92.1)
Fail	9 (7.9)

**Table 2:** USMLE and ITE scores of residents who passed ABIM-CE versus those who did not

	Scores (Mean (SD))		*p*-value
	ABIM-CE Pass	ABIM-CE Fail	
USMLE Step 1	231 (18)	209 (12)	< 0.001
USMLE Step 2	233 (19)	204 (19)	< 0.001
USMLE Step 3	208 (13)	200 (6)	0.07
Third-year ITE	69 (7)	59 (6)	< 0.001

*ABIM-CE* American Board of Internal Medicine- Certifying Exam, *ITE* In-Training Exam, *SD* Standard Deviation, *USMLE* United States Medical Licensing Exam

All residents (*n* = 9) who failed ABIM-CE scored < 220 in USMLE Step 1 which was 25% of total residents with USMLE Step 1 score of < 220 (*n* = 35). Among 31 residents with USMLE Step 2 CK score < 220, 20% (*n* = 6) failed ABIM whereas only 10% (*n* = 3) with USMLE Step 2 CK score > 220 failed ABIM-CE. Similarly, 9% of residents with USMLE Step 3 score < 220 failed ABIM-CE; all residents who failed had scored < 220.

First, logistic regressions were employed to predict ABIM-CE passing with USMLE Step 1, USMLE Step 2 CK, USMLE Step 3, and ITE tests separately controlling for gender, country of the medical school, and 1 year of the gap between medical school and the start of residency. There was a significant correlation of passing ABIM-CE with 10 points increase in USMLE Step 1 (Odds ratio [OR] 2.70; 95% Confidence Interval [CI] 1.38–5.29) and 10 points increase in Step 2 CK (2.31; 95% CI 1.33–4.01). However, a 10 points increase in Step 3 (OR 1.63; 95% CI 0.81–3.29) did not significantly predict passing ABIM-CE. A 5% increase in ITE percentage increased the likelihood of passing ABIM-CE (OR 3.89, 95% CI 1.68–8.98).

Next, we calculated the average of USMLE Step scores and predicted the ABIM-CE pass result using the average Step scores. Table 3 shows the results from the analysis of maximum likelihood estimates. A 10 points increase in average Step scores will lead to a higher chance of passing ABIM-CE tests (OR = 2.96, 95% CI = 0.95–9.20)) but not significantly at α = 0.05 level. There was no relationship between ABIM-CE results and gender, the country of medical school (American medical graduate = AMG vs. International medical graduate = IMG). The increase in the gap (1 year) was not significantly associated with the chances of passing ABIM-CE (OR 0.82; 95% CI 0.364–1.06) (Table 3).

**Table 3:** Odds of passing ABIM-CE in relation to demographic characteristics and scores

Variables	Analysis of maximum likelihood estimates	
	Odd’s ratio	*p*-value
Mean of USMLE Step 1, Step 2, & Step 3 (10 points)	2.961	0.0605
Gap between medical school and residency (1 year)	0.822	0.1231
Third-year- ITE percentage (5 points)	2.916	0.0240*
Gender of the residents (Female vs. Male)	2.754	0.3038
Medical school country (AMG vs. IMG)	4.969	0.2694

*AMG* American Medical Graduate, *IMG* International Medical Graduate, *ITE* In-Training Exam, *USMLE* United States Medical Licensing Exam

* significant with *p*-value < 0.05

**Fig. 1:**
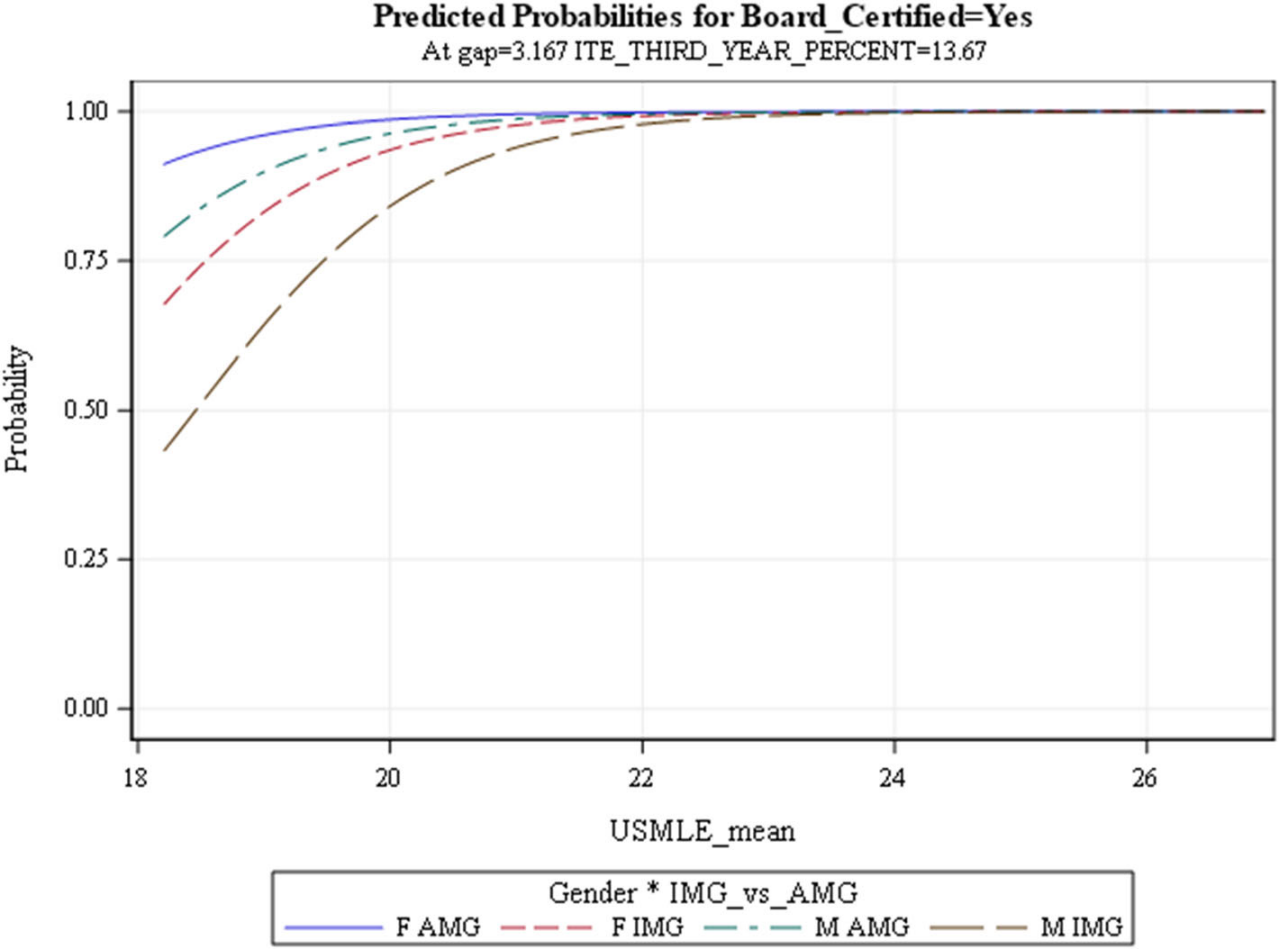
Predicted probability curve for passing ABIM-CE

**Fig. 2:**
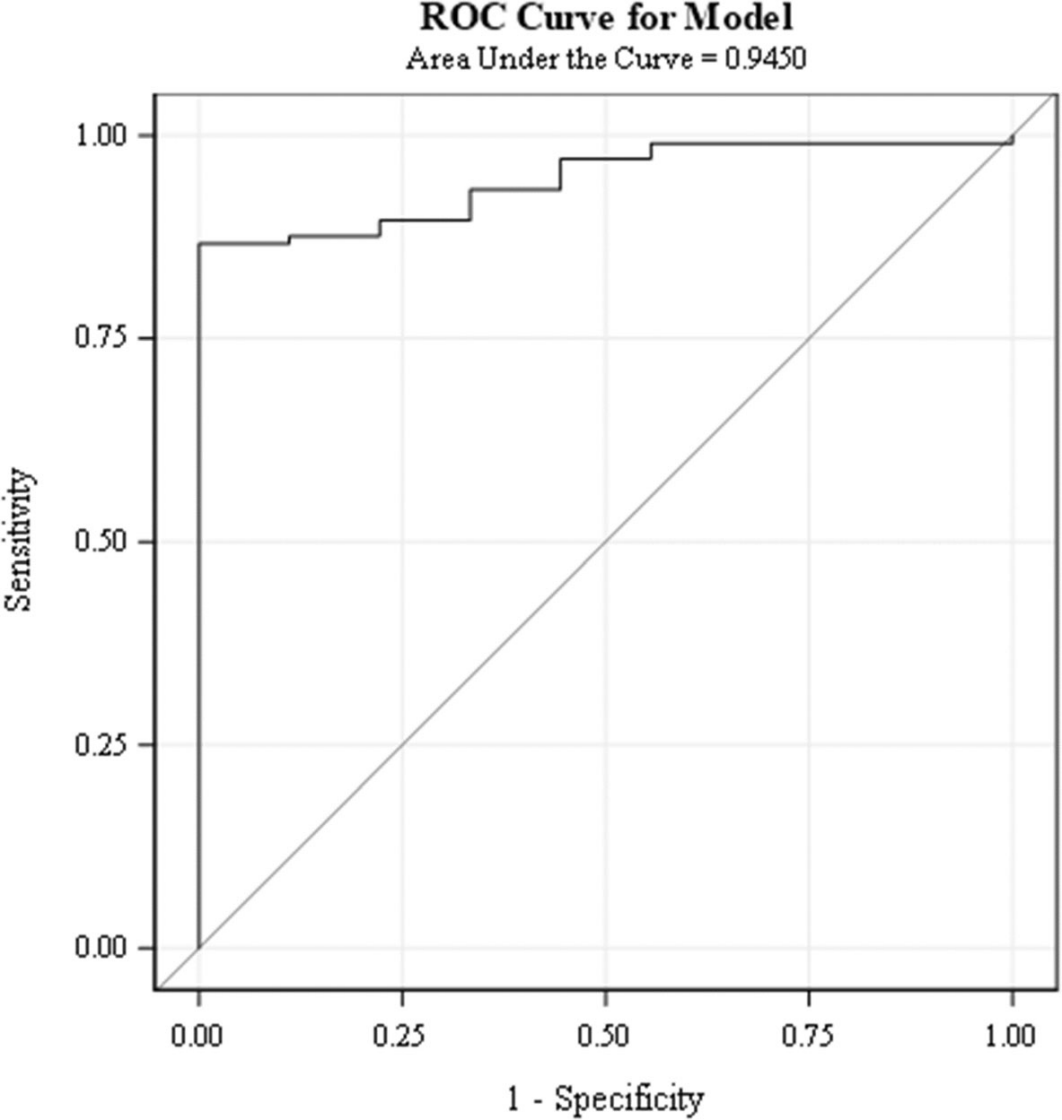
ROC curve

The probability curve (with third year-ITE percentage and the gap set at mean values) predicted that the chance of passing ABIM- CE was around 80% with USMLE scores higher than 200 and ABIM-CE passing chance increased to almost 100% with USMLE scores of 240 or higher (Fig. 1). A ROC (receiver-operating characteristic) curve was computed to assess the accuracy of the model’s ability to predict passing the ABIM-CE. The area under the ROC curve was 0.945 (Fig. 2), which indicates that the probability of our model will rank a randomly chosen “pass the board” higher than “not pass the board” is 0.945.

We repeated the analysis using standardized USMLE step scores and standardized ITE test scores with mean at zero and standard deviation (SD) at 1. One SD increase in USMLE Step 1 lead to a significantly higher chance of passing ABIM-CE (OR = 6.41, 95% CI 1.84–22.46) and one SD increase in USMLE Step 2 CK lead to a significantly higher chance of passing ABIM-CE (OR = 5.62, 95% CI 1.80–17.60). However, a 1 SD increase in USMLE Step 3 is not significantly associated with passing ABIM-CE (OR = 1.89, 95% CI 0.76–4.68). Table 4 shows the results with a 1 SD increase in average USMLE score and ITE percentage increase after controlling for demographics. Our analysis also found that a 1 SD increase in the ITE test leads to a higher chance of passing ABIM (OR = 5.0, 95% CI 1.24–20.25). The results indicate that one SD change in the ITE test has the most significant effect on increasing the chance of passing the ABIM-CE.

**Table 4:** Odds of passing ABIM-CE in relation to demographic characteristics and standardized scores

Variables	Analysis of maximum likelihood estimates	
	Odd’s ratio	*p*-value
Mean of USMLE Step 1, Step 2, & Step 3 (1 SD increase)	4.686	0.0605
Gap between medical school and residency (1 year)	0.822	0.1231
Third-year- ITE percentage (1 SD increase)	5.004	0.0240*
Gender of the residents (Female vs. Male)	2.754	0.3038
Medical school country (AMG vs. IMG)	4.969	0.2694

* significant with *p*-value < 0.05

## Discussion

Our analysis showed that USMLE Step 1, USMLE Step 2 CK, USMLE Step 3, and third-year ITE scores have a predictive value in determining the chances of passing ABIM-CE. Among them, ITE percentage is the most predictive, followed by USMLE Step 1, USMLE Step 2 CK, and USMLE step 3 in the descending order.

Residency programs use USMLE scores as one of the initial screening tools to select their potential candidates from residency applicants. USMLE comprises of three steps - USMLE Step 1, USMLE Step 2, and USMLE Step 3, to evaluate the appropriate use of medical knowledge in patient care by the examinees. The USMLE Step 2 exam consists of two sections: USMLE Step 2 CK (Clinical Knowledge) and USMLE Step 2 CS (Clinical Skills). Currently, USMLE Step 1, USMLE Step 2 CK, and USMLE Step 3 are MCQ based exams and they reports score in a three-digit format [13]. USMLE Step 2 CS tests their clinical skills, and reports only pass or fail [13]. Previously, USMLE reported scores in percentile format. However, in 1999, the percentile-based system was eliminated in favor of a three-digit and two-digit scaled scoring system. USMLE removed the two-digit score from the score report in April 2013.

USMLE exam scores range anywhere from 1 to 300, and most examinees score between 140 to 260. USMLE Step 1 passing score is 194; the national mean approximately 229 (sd = 20) [13]. According to the National Resident Matching Program (NRMP), the mean score for US allopathic candidates matching into residency programs in 2016 was 233.2 (sd = 17.4) [13]. For the 2013–2014 and 2014–2015 academic years, the mean USMLE Step 2CK score is 240 (sd = 18) for first-time takers from the medical schools in the United States and Canada. The minimum passing score for students taking USMLE Step 2 CK after July 1, 2014, is 209 and was 209, at least until May 2018 [13]. The minimum USMLE Step 3 passing score will be raised to 198 from 196 from the beginning of January 1, 2020 [13].

 USMLE Step 1 score was included in a predictive tool to rank applicants after the residency interview, the other factors in the tool were medical school quality, overall medical school performance, and performance in junior medicine clerkship [14]. However, there is limited data to prove that USMLE scores have a strong correlation with the performance in ABIM-CE [12]. The results from our study showed that the USMLE Steps scores can predict the ABIM-CE results, USMLE step 1 score more predictive than USMLE Step 2 CK scores. The chance of failing ABIM-CE is higher with USMLE Scores below 220, more so with USMLE Step 1 than USMLE Step 2 CK or USMLE Step 3. Kay et al. reported a modest correlation between USMLE Step 1 and ABIM-CE scores [13]. There are reports of consistent results showing a correlation between USMLE scores and the results of the certification examination in various specialties and subspecialties [15–19]. Higher USMLE Step 1, USMLE Step 2 CK, and composite scores were also associated with better performance on Emergency Medicine boards, with USMLE Step 2 scores being the strongest predictor [18]. Lower USMLE Step 1 score was previously found to be predictive of failing certifying exams in surgery and pediatrics [19, 20]. 

Performance in ITE is another tool used to predict the ABIM-CE results. ITE scores are expected to improve from the first year to the third year in IM residency training programs [13]. Previous ITE scores are strongly associated with subsequent ITE than USMLE scores [10]. ITE scores are useful for residency programs to recognize residents needing assistance or interventions from the program to increase their chances to pass the ABIM-CE. Since the third year-ITE is the exam closest to the ABIM-CE, the results of the third year ITE can be used to gauge the readiness of a resident to take ABIM-CE. In our study, third year-ITE percent had a positive correlation with ABIM-CE passing chance, the correlation was even better than USMLE scores. Similarly, a previous study by Kay et al. had found a modest correlation between ITE and ABIM-CE scores [13]. Also, reports suggest that residents scoring in the bottom quartile on their ITE were at increased risk of failing boards while those scoring in the top quartile of the ITE had a 100% pass rate [13]. In the past, a few other studies examined the association between ABIM-CE results and ITE scores with similar results [10, 21–23]. Babott et al. reported that second-year ITE scores of more than 61% predicted a 100% pass rate in ABIM-CE with 41% sensitivity and 100% specificity [7]. Brateanu and colleagues developed a nomogram to predict the ABIM-CE performance, which included the ITE scores of each year and the number of overnight calls in the last 6 months of residency [15], and their analysis reported that the third year-ITE was the most important predictor of chances to pass ABIM-CE. Univariate analysis showed a good correlation of USMLE results with ABIM-CE; however, multivariate regression did not show a statistically significant correlation between USMLE and ABIM-CE results.

 The predictive value of ITE scores with the performance in certifying exams has been reported in other specialties as well [19, 24–26]. A study in Emergency Medicine showed that third year-ITE scores were most predictive of the score in the certification exam [25]. Similarly, a low score in ITE at any time during residency increased the chances of failing a certification exam in surgery [19].

Passing in ABIM-CE is of utmost importance to residents [27], and their residency training programs as well. ACGME requires a first-attempt examinee pass rate of at least 80% for continued accreditation of a residency program. Hence, residency programs put a great deal of effort to choose residents with strong clinical skills and ones likely to perform well in ABIM-CE. Residency programs hold practice exams, teaching sessions, recommend mandatory usage of Question banks, and encourage enrollment in Board Review Courses to enhance the ABIM-CE passing rates.

A majority of the IMGs, unlike AMGs, tend to complete their USMLE Step exams after completion of their medical school curriculum. During their preparation for USMLE exams, IMGs pursue observerships to familiarize themselves with the United States medical system and to improve their clinical skills. Several candidates interested in pursuing an academic career spend time doing research, and a few enroll in  master’s programs after completion of medical school and prior to applying for residency. Thus, IMG applicants  generally have a gap of 1 to 3 years between the completion of medical school and beginning residency. Brateanu et al. reported a weak positive correlation between the length of the gap and the performance in ABIM-CE [16]. Another study on IMGs by Kanna and colleagues showed that the gap between medical school and residency was not significant in predicting ITE scores [28]. Similarly, our study did not show any association of 1-year gap between medical school and starting residency with the chances of passing ABIM-CE . Also, gender differences and place of medical school (AMG vs. IMG) were not associated with ABIM-CE results.

Our study has a few limitations. First, this is a single-center study performed using data from previous residents at a small training program. Second, there is no tool to calculate the equivalence of COMLEX to USMLE, so we did not include osteopathic residents in our analysis and we could not compare ABIM-CE results of osteopathic residents with allopathic residents. Third, there is minimal variation in the ABIM-CE pass rates over the last few years since nearly all of the candidates in our sample passed ABIM-CE. Finally, starting from January 1, 2022, USMLE plans to report USMLE Step 1 as pass or fail instead of the current three digit scoring. After the implementation of the pass or fail to report USMLE Step 1 results, we need to determine if USMLE Step 1 results or the number of attempts to pass the exam has any effect on the results of ABIM-CE. 

We need to consider the predictive ability of USMLE Step 1, USMLE Step 2CK and 3rd year ITE scores in identifying residents at risk for failing ABIM-CE to implement early interventions or remediations plans to enhance their chances of passing ABIM-CE. Some of the remediation plans include enrolling in board review courses, attending conferences, or self-study courses, however, their efficacy in helping residents pass the ABIM-CE is unclear [29]. Although there are reports of improved ABIM-CE results with a directed reading program and individual education plan, although literature regarding the efficacy of these strategies is limited [30, 31]. At our program, we identified residents with scores < 35 percentile in the PGY3 ITE exam, and encouraged them to enroll in board review courses or use one of the available Question bank's which yielded a 100% ABIM-CE pass rate over the past few couple of years. Few residents reported that joining group discussions or studying with a partner was beneficial in addition to enrolling in board review courses or using Question banks. Furthermore, several other unexplained factors may determine whether a resident passes or fails the ABIM-CE.

## Conclusion

The third-year ITE score is more predictive of ABIM-CE results compared to USMLE Step 1 and USMLE Step 2CK scores. Both USMLE Step 1 and Step 2 CK scores > 220 are independent predictors of success in passing ABIM-CE as well, but USMLE Step 1 score is more predictive compared to USMLE Step 2CK score. Thus, programs can identify internal medicine residents at risk of failing ABIM-CE and implement measures, such as enrolling them in question banks or board review courses to improve their chances of passing ABIM-CE.

## Data Availability

We have provided the raw data as a supplemental file.

## Abbreviations

ABIM: American Board of Internal Medicine
ABIM-CE: American Board of Internal Medicine Certifying Exam
COMLEX: The Comprehensive Osteopathic Medical Licensing Examination
IM: Internal Medicine
ITE: In training exams
ROC: Receiver-operating characteristic
USMLE: United States Medical Licensing Examination
